# Cardiovascular medicine in China: what can we do to achieve the Healthy China 2030 plan?

**DOI:** 10.1186/s12916-018-1133-4

**Published:** 2018-08-24

**Authors:** Yihua Bei, Tingting Yang, Junjie Xiao

**Affiliations:** Institute of Cardiovascular Sciences, Shanghai University, 381 Nan Chen Road, Shanghai, 200444 China

**Keywords:** Cardiovascular diseases, China, Healthy China 2030

## Abstract

Cardiovascular diseases (CVDs) represent the leading cause of death in China. The Chinese government approved the Healthy China 2030 plan (*jiànkāng zhōngguó 2030*), emphasizing the strategic role of health in China’s development. As morbidity and mortality from CVDs are constantly increasing in China, the prevention and treatment of CVDs are vital to achieve this plan. Following the major principles of health priority, science and technology innovation, scientific development, and balanced medical resource allocation outlined in the Healthy China 2030 plan, this Commentary briefly introduces the current status of CVDs in China and marks the important events undertaken to achieve this plan.

## Background

With the rapid socioeconomic development in China, environmental issues and changes in lifestyle and urbanization have brought a series of new challenges in maintaining and promoting people’s health. Cardiovascular diseases (CVDs) represent the leading cause of death in China, accounting for two out of five deaths [1]. As published in the official Report on Cardiovascular Diseases in China 2017 by the National Center for Cardiovascular Diseases, the total number of CVD patients in China in 2017 was 290 million [2]. A rapid aging process will further increase the prevalence and mortality rate of CVDs in China [3].

In 2016, the Chinese government approved the Healthy China 2030 plan (*jiànkāng zhōngguó 2030*), emphasizing the strategic role of health in China’s development and outlining the major principles to achieve this, including health priority, science and technology innovation, scientific development, and balanced medical resource allocation. As morbidity and mortality from CVDs are constantly increasing in China [2], the prevention and treatment of CVDs have become vital to achieve this plan. Taking the health of the whole people as the fundamental purpose, China is also making efforts to promote science and technology innovation, and to develop balanced medical resource allocation over the country. Following the major principles of the Healthy China 2030 plan, this Commentary briefly introduces the current status of CVDs in China and marks the important events undertaken to achieve this plan.

## Health priority

This principle aims to integrate the concept of health in the development of all strategic policies in China. Efforts must be made to promote a healthy lifestyle in people of all ages [4], and to tackle environmental issues to ensure the sustainable development of health and society. The China Cardiovascular Health Index (2017) is the world’s first national level evaluation on CVD prevention and control (http://cvindex.hsmap.com), providing official data on the prevalence, treatment, and public health policies and services for CVDs, as well as the scientific statistics of exposure and control of cardiovascular risk factors. These include blood pressure, dyslipidemia, diabetes mellitus, smoking, overweight and obesity, sedentary lifestyle, unreasonable diet, and air pollution. For instance, fine particulate matter (≤ 2.5 μm in aerodynamic diameter; PM2.5) pollution is a serious environmental issue in China [5]. Long-term exposure to PM2.5 over certain concentrations has been proven to be associated with ischemic cardiac disease and hypertension [6, 7]. Although the PM2.5 concentration had declined by 6.5% in 2017 compared to 2016, a stricter control of air pollution is being implemented to achieve better surveillance and assessment of air quality, including emergent strategies such as reducing pollution originating from motor vehicles, coal burning, industrial production, and outdoor activities which create dust [8].

## Science and technology innovation

With great efforts in science and technology innovation, novel techniques and methods have been developed in the management of CVDs. China has 11 million coronary arterial disease (CAD) patients, and statistics released from the China Cardiovascular Intervention Forum 2018 (http://ccif.icirculation.com) reported that cardiovascular interventional therapy was provided to 753,142 patients (~ 1.5 stent per case) in 2017. The Chronic Total Occlusion Club, China, founded in Shanghai in 2005, aims to recruit top Chinese professional cardiac interventional physicians to perform, demonstrate, and discuss the high standard percutaneous coronary intervention (PCI) for patients with chronic total occlusion (CTO) and to collect clinical data from over 50 centers and more than 80 CTO PCI physicians (Chronic Total Occlusion Club, China Registry Study). This is expected to further improve interventional therapy and the long-term prognosis of CTO patients. Chinese governmental and non-governmental grants have also been invested on clinical research for CADs, focusing on the efficacy and safety of antithrombotic therapy and the angiographic efficacy after PCI [9–14]. New data from clinical research may lead to the update of guidelines and improve the clinical management of CADs.

Despite advances in basic research for CVDs, research and development for original new drugs remains underdeveloped in China. Conversely, research and development of novel medical materials and devices is rapidly advancing. Major comorbidities of heart failure (HF) in China have gradually shifted from valve heart diseases to CADs and hypertension [15]. To date, cardiac transplantation remains the only effective therapy for end-stage HF patients despite advances in HF management. Nevertheless, only approximately 300 heart transplant surgeries are performed annually in China due to the limited availability of donated organs. The clinical use of total artificial heart and ventricular assist devices (VADs) is an effective bridge to cardiac transplantation, as well as an alternative therapy for acute and end-stage HF patients when a donor heart is unavailable. Benefitting from China’s independently developed, third generation magnetic-levitation CH-VAD, three patients with terminal stage HF successfully received VAD therapy following approval through the humanitarian device exemption policy in Fuwai Hospital, Beijing, in 2017. The National Center for Cardiovascular Diseases reported that all three patients survived well, with one patient eventually finding a donor transplant 192 days after having received VAD. Thus, the CH-VAD should be able to fill the gap in the field of domestic artificial hearts in China.

Chinese scientists have also developed superaligned carbon nanotube heart patches to guide oriented cardiomyocyte growth and promote electrophysiological homogeneity for engineered cardiac tissues [16]. The superaligned carbon nanotube-based, one-piece pacemaker electrode may also be potentially applied for cardiac resynchronization therapy in myocardial infarction patients with HF [16]. We hope that these Chinese-developed medical materials and devices will soon be approved for clinical trials and may one day provide new hope for HF patients.

## Scientific development

Scientific development refers to the comprehensive implementation of medical services. Data from a nationally representative sample of 96,121 Chinese adults aged 20 years or older reported that the estimated percentage of ideal cardiovascular health in adults was extremely low (0.1% in men and 0.4% in women) [17]. The guideline for CVD prevention in China highlights the priority of prevention and stipulates primary and secondary prevention strategies for CVDs. In 2016, the China Cardiovascular Association launched a project for the systemic implementation of chest pain centers in hospitals (http://www.chinacpc.org), aiming to promote early diagnosis and timely therapy for patients with CADs, aortic dissection, pulmonary embolism, or other critical illnesses. In recent years, cardiac rehabilitation, mainly consisting of medical and lifestyle interventions, has also attracted attention due to improved recovery and outcomes in CVD patients [18]. The number of cardiac rehabilitation centers has rapidly grown to approximately 500 by 2017. Recently, a Modern Cardiac Rehabilitation Center was established in Shanghai, aiming to perform high standard external counterpulsation and cardiac shockwave therapy, and to employ precise D-SPECT cardiac imaging to evaluate the therapeutic effect. Moreover, concepts such as the ‘Better Life with a Stent’ slogan have been established to promote communication between patients and clinicians, and to encourage their participation and follow-up in cardiac rehabilitation.

## Balanced medical resource allocation

China is the most populous country worldwide. Hospitalization expenses for acute myocardial infarction reached 15.34 billion yuan (2.31 billion USD) in 2015, with the annual total medical costs for CVDs quickly increasing [1]. However, medical resources are not equally available in all regions of the country due to the variations in economic and social development. Over the last decade, China underwent a significant health system reform, increasing the presence of health services in rural areas. Experienced clinicians are encouraged to work in rural hospitals for a certain period to promote improved disease diagnosis and treatment. Further, gradual implementation of provincial control of pension insurance planning will help attain a fair and balanced healthcare system for all.

In summary, China is undergoing great efforts to promote a healthy lifestyle and better interventions for CVDs, providing great hope for the achievement of the Healthy China 2030 plan.
